# Evaluation of the efficacy and feasibility of concurrent weekly docetaxel-nedaplatin and hypo-fractionated radiotherapy in atypical histologic subtypes of primary and metastatic mediastinal malignancies

**DOI:** 10.3389/fonc.2022.974394

**Published:** 2022-10-07

**Authors:** FanJun Meng, XinLei Ai, Bin Wang, Yin Zhou, Su Li, DaQuan Wang, FangJie Liu, NaiBin Chen, Rui Zhou, JinYu Guo, XiaoYan Huang, ShaoHan Yin, Bo Qiu, Hui Liu

**Affiliations:** ^1^ Department of Radiation Oncology, Jieyang People’s Hospital (Jieyang Affiliated Hospital, Sun Yat-sen University), Jieyang, China; ^2^ Department of Radiation Oncology, Sun Yat-sen University Cancer Center, Guangzhou, China; ^3^ State Key Laboratory of Oncology in South China, Sun Yat-sen University Cancer Center, Guangzhou, China; ^4^ Collaborative Innovation Center for Cancer Medicine, Sun Yat-sen University Cancer Center, Guangzhou, China; ^5^ Evidance Medical Technologies Inc., Suzhou, China; ^6^ Department of Clinical Research, Sun Yat-sen University Cancer Center, Guangzhou, China; ^7^ Department of Radiology, Sun Yat-sen University Cancer Center, Guangzhou, China

**Keywords:** mediastinal malignancy, concurrent chemoradiotherapy, in-field locoregional progression-free survival, LQRGC/TCP model, hypo-fractionated radiotherapy

## Abstract

**Background:**

We aimed to evaluate the efficacy and feasibility of concurrent weekly docetaxel-nedaplatin and hypo-fractionated radiotherapy (hypo-RT) in atypical histologic subtypes of primary and metastatic mediastinal malignancies.

**Methods:**

Fifty-four patients diagnosed with atypical primary or metastatic mediastinal malignancies were retrospectively reviewed. 30 patients received concurrent weekly docetaxel and nedaplatin and hypo-RT (CChRT group) and 24 patients had hypo-RT alone (hRT group). Overall response rate (ORR), in-field locoregional progression-free survival (LPFS) and toxicities were analyzed. The radiobiological effect was evaluated by the LQRGC/TCP model, incorporating four “R”s of radiobiology, Gompertzian tumor growth and radio-sensitizing effect of chemotherapeutic agent. The biologically effective doses (BEDs) were calculated.

**Results:**

The median follow-up time was 29.2 months for all patients. The ORR was 86.7% in CChRT group, compared with 62.5% in hRT group (*p*=0.033). The 2-year in-field LPFS of CChRT and hRT group was 73.4% and 47.3%, respectively (*p*=0.003). There was no significant difference of any >=Grade 3 toxicities between the two groups (*p*=0.754). The mean total dose and mean BED by the LQRGC/TCP model in CChRT group were 58.2Gy and 72.34Gy, versus 52.6Gy and 67.25Gy in hRT group.

**Conclusions:**

Concurrent weekly docetaxel-nedaplatin and hypo-RT achieved promising in-field LPFS and tolerable toxicities compared with hypo-RT alone in different histologic subtypes of primary and metastatic mediastinal malignancies.

## Introduction

The primary and metastatic mediastinal malignancies of atypical histology were considered relatively radiation-resistant and lacked of effective local treatment except for surgery (1–3). Recent studies suggested that hypo-fractionated radiotherapy (hypo-RT) could improve tumor control by increasing the biologically effective dose (BED) and shortening treatment time (4). However, dose escalation remained difficult for the high incidence of radiation-induced toxicities to critical organs such as trachea, esophagus and heart. Therefore, adding concurrent chemotherapy to hypo-RT might offer better treatment outcome for this sub-group of patients. Recently, several studies suggested that hypo-RT with concurrent chemotherapy showed efficacy in a variety of malignancies, including uterine sarcoma, osteogenic sarcoma and colorectal carcinoma (5–7). Patients with atypical histologic subtypes of primary and metastatic mediastinal malignancies could benefit from hypo-RT combined with concurrent platinum-based chemotherapy.

Taxanes plus platinum are commonly administrated concurrently with radiotherapy in many cancer types. Compared with paclitaxel, docetaxel demonstrates superior pharmacological prosperities, including higher cytotoxicity and broader tissue distribution. Preclinical studies reported that docetaxel demonstrated a potent radio-sensitizing effect that is 10 times that of paclitaxel at equivalent concentration (8). Our previous studies also showed that docetaxel administrated at 25mg/m^2^ weekly had superior radio-sensitizing effect than paclitaxel at 50mg/m^2^ weekly (chemo-induced BED: 2.52 vs. 1.89Gy) (9). Low dose concurrent docetaxel and nedaplatin (DP) showed a promising radiosensitizing effect on in-field disease control with 1-year LPFS of 66.7%-83.3% in esophageal cancer (10). In breast cancer, Koukourakis et al. reported a 2-year PFS of over 80% with accelerated hypo-RT with concurrent docetaxel-based chemotherapy (11). Our previous studies found that hypo-RT with concurrent weekly docetaxel-platinum regimen could achieve promising locoregional control with tolerable pulmonary toxicity in LANSCLC (12, 13). We also established an extended LQRGC/TCP model, which fits well with locoregional progression free survival (LPFS) in LANSCLC treated with concurrent chemoradiotherapy compared with LQ/TCP model (9).

In this study, the in-field local tumor control was retrospectively analyzed in patients with atypical histologic subtypes of primary and metastatic mediastinal malignancies treated with hypo-RT and concurrent weekly DP or hypo-RT alone based on long-term computed tomography (CT)/magnetic resonance imaging (MRI) follow-up. We aimed to evaluate the efficacy and feasibility of concurrent weekly docetaxel-nedaplatin and hypo-fractionated radiotherapy in atypical histologic subtypes of primary and metastatic mediastinal malignancies. An extended tumor control probability (TCP) model was employed for further validation on the radio-sensitizing effect of the docetaxel-platinum combination.

## Materials and methods

### Patients

We retrospectively evaluated 54 patients with unusual primary and metastatic mediastinal malignancies in our center between April 2015 to January 2020. There were 30 patients treated with hypo-RT and concurrent weekly DP (CChRT group) and 24 patients treated with hypo-RT alone (hRT group). The eligibility criteria were as follows: 1. age 18 to 75 years old; 2. histologically confirmed thoracic malignancies, including primary mediastinal malignancies and oligometastatic pulmonary lesions; 3. Eastern Cooperative Oncology Group (ECOG) performance status (PS) score of 0-2; 4. regular CT and/or MRI scan till disease progression; 5. Life expectancy >6 months.

This study was reviewed and approved by the ethics committee of Sun Yat-sen University Cancer Center. Written informed consent for the use of clinical data was obtained from all patients.

### Treatment

Patient immobilization, simulation, and treatment planning were conducted according to the standard protocol of radiotherapy for thoracic cancer in our center (14). All patients were immobilized in the supine position using a vacuum cradle. The simulation CT sets were scanned from the Atlas level to the second lumbar vertebra level with 5mm thick slices, using free-breathing 4-dimensional (4D) CT scan, and then a maximum intensity projection (MIP) set was constructed for target contouring. The gross tumor volume (GTV) comprised primary disease and positive lymph nodes for primary thoracic malignancies, or thoracic metastasis diagnosed by biopsy, CT scan, or positron emission tomography/computed tomography (PET/CT) scan. The GTVs were composite volumes from CT scans of 10 respiratory phases. The planning target volume (PTV) covered GTV plus an expansion of 6 mm.

Hypo-RT was delivered using the intensity-modulated radiotherapy (IMRT) technique with a total dose of 40-69Gy in 5-23 daily fractions (3-7Gy per fraction). For patients at a high risk of toxicities (bulky tumor, or tumor adjacent to esophagus or spinal cord), split-course hypo-RT was delivered with an interval of 4 weeks between two courses. In that case, re-planning was performed to adapt the dose to residual disease in the second course. It was required that at least 95% of the PTV-GTV received 95% of the prescription dose. Image guidance with daily cone-beam CT prior to treatment was used for all patients.

Patients in the CChRT group received concurrent docetaxel 25 mg/m^2^ intravenously (IV) plus nedaplatin 25 mg/m^2^ IV weekly. Doses were adjusted according to toxicities during the treatment.

### Follow-up

Patients were followed up with regular history taking, physical examination and imaging surveillance including chest and upper abdomen CT, and brain MRI. PET/CT scan, bone scan, and biopsy were performed if necessary. Evaluation of response by chest and abdomen CT was first carried out 8 weeks after the completion of hypo-fractionated radiotherapy. Routine surveillance imaging was conducted till disease progression or death. Tumor response was evaluated by Response Evaluation Criteria in Solid Tumors (RECIST) version 1.1. Complete remission (CR) was defined as disappearance of all target lesions. Partial remission (PR) was defined as at least a 30% decrease in the sum of diameter of target lesions. Progressive disease (PD) is defined as an increase of more than 20% in the size of the longest diameter of a target tumor from the baseline. Stable disease (SD) is defined as fitting the criteria neither for PD nor a PR. In-field overall response rate (ORR) and disease control rate (DCR) were assessed according to in-field tumor shrinkage, despite of the status of out-field lesions. In-field ORR was defined as the proportion of patients who achieve a CR or PR. In-field DCR was defined as the proportion of patients who achieve a CR, PR or SD. In-field duration of response (DOR) was measured from the time at which CR or PR are first met until the first date of in-field PD or death. In-field LPFS was defined as the time from the start of radiotherapy to the date of in-field locoregional progression or the date of last follow-up before September 1st, 2020. The treatment related toxicities were evaluated according to Common Terminology Criteria for Adverse Events Version 5.0 (CTCAE 5.0).

### Statistical analysis

Descriptive statistics are used to summarize demographics and safety. Actual survival curves were generated using the Kaplan-Meier method, which was used to assess in-field LPFS over time. The clinical endpoint for model prediction was in-field LPFS. Descriptive statistics and survival analysis were performed with SPSS 26.0 software (IBM Corp). Statistical tests were based on a two-sided significance level. A value of *P*<0.05 was considered statistically significant.

### Tumor control probability modeling

#### LQRGC model

This study employed the LQRGC model and its associated TCP model to account for the joint effects of radiotherapy and chemotherapy (9). The construction of LQRGC/TCP model is briefly presented below.

The survival fraction (SF) of tumor cells in this model is expressed as:


(1) $$SF\left(t\right)=\text{exp}\left(-\alpha D-\beta G\left(\tau_{R}\right)D^{2}+\left(\frac{1}{2}\sigma^{2}\right)G\left(\tau_{s}\right)D^{2}+\text{ln}2\frac{T-T_{k}}{T_{P}}+{\left(\text{ln}2\frac{t}{T_{P}}\right)}^{\delta }-C_{i}\right)$$

As detailed in our previous study (15, 16), the generalized Lea-Catcheside function *G(τ)* takes the form as below:


(2) $$G\left(\tau \right)=\left(\frac{2}{D^{2}}\right)\int_{0}^{\text{T}}R\left(u\right)du\int_{0}^{u}R\left(w\right)\text{exp}\left(-\frac{u-w}{\tau }\right)dw$$

The detailed model parameters were described in **Supplementary 1**. The function *R*(*t*) to simulate the hypo-fractionated dose schedule is expressed by pulse trains (**Supplementary 2.1**):

*P*(*t*) is the single pulse function as shown in **Supplementary 2.1**, *T_i_*is the beginning of treatment time in fraction *i*. Otherwise, the non-zero period of *P(t)*, which is assumed to be 10 minutes in this study, is used to simulate the treatment time in each fraction.


(3) $$R\left(u\right)=\sum_{\text{i}=0}^{\text{n}}P\left(t-T_{i}\right)$$

The TCP function derives from Tai et al.'s (17) study:


(4) $$TCP\left(t\right)=1-\frac{1}{\sqrt{2\pi }}\int_{-\infty }^{x_{0}}\text{exp}\left(-\frac{x^{2}}{2}\right)dx$$

The parameters were detailed in **Supplementary 1.2**.


(5) $$x_{0}=\frac{\bar{K}-K_{cr}}{\sigma_{k}}=\frac{SF-K_{cr}/K_{0}}{\sigma_{k}/K_{0}}$$

As detailed in our previous study (9), BED calculated by the LQRGC model is expressed as:


(6) $$BED=\left(D+\frac{\beta G(\tau_{R})D^{2}}{\alpha }-\frac{\frac{1}{2}\sigma^{2}G(\tau_{S})D^{2}}{\alpha }-\frac{\text{In}2\left(T-T_{k}\right)}{\alpha T_{P}}+\frac{C_{i}}{\alpha }\right)$$

#### Model optimized procedures

The initial LQRGC model parameters were taken from those of NSCLC and the chemotherapy regimen of DP (**Table 4**). The optimized model parameters were determined using a gradient descent method by minimizing the mean absolute error, constituting the new LQRGC model. Confidence intervals (CIs) for the parameters were calculated by nonlinear regression. All the aforementioned calculations in tumor control probability modelling were conducted on a cloud-based clinical data service platform iRAAS^®^v2.0 (Homology Medical, Ningbo, China, 2020).

## Results

A total of 54 patients with atypical histologic subtypes of primary and secondary mediastinal malignancies treated by hypo-RT with concurrent DP regimen (CChRT group) or hypo-RT alone (hRT group) were analyzed. The baseline characteristics and treatment-related parameters are presented in **Table 1**. There were 12(40.0%) patients with primary malignancies and 18(60.0%) patients with oligo-metastatic tumors in the CChRT group, compared with 11(45.8%) patients with primary thoracic malignancies and 13 (54.2%) patients with oligo-metastatic tumors in the hRT group (*p*=0.667). The details of pathological types are shown in **Table 1**. There was no significant difference in baseline characteristics between the two groups.

**Table 1:** Baseline Characteristics (n=54).

Characteristic	CChRT group (n=30)	hRT group (n=24)	*P*
	Value or No. (%)	Value or No. (%)	
**Age**			
Median	51.0	48.0	0.462
Range	25~75	20~75	
**Sex**			
Male	18 (60.0)	14 (58.3)	0.901
Female	12 (40.0)	10 (41.7)	
**ECOG PS**			
0-1	29 (96.7)	24 (100.0)	0.367
2	1 (3.3)	0 (0.0)	
**Primary mediastinal malignancies**	12 (40.0)	11 (45.8)	0.667
Thymic carcinoid	1 (3.3)	0 (0)	
Thymic atypical carcinoid	4 (13.3)	1 (4.2)	
Thymic undifferentiated carcinoma	1 (3.3))	1 (4.2)	
Thymic squamous cell carcinoma	1 (3.3)	1 (4.2)	
Thymic basaloid carcinoma	0 (0)	1 (4.2)	
Mediastinal sarcomatoid carcinoma	1 (3.3)	0 (0)	
Mediastinal mucinous adenocarcinoma	1 (3.3)	0 (0)	
Mediastinal germ cell tumor	1 (3.3)	4 (16.7)	
Tracheal NUT carcinoma	1 (3.3)	0 (0)	
Tracheal adenoid cystic carcinoma	1 (3.3)	0 (0)	
Diaphragm epithelioid mesothelioma	0 (0)	1 (4.2)	
Mediastinal classical Hodgkin lymphoma	0 (0)	1 (4.2)	
Diffuse large B-cell lymphoma	0 (0)	1 (4.2)	
**Primary tumor of pulmonary metastasis**	18 (60.0)	13 (54.2)	0.667
Parotid acinar cell carcinoma	1 (3.3)	0 (0)	
Hepatocellular carcinoma	4 (13.3)	0 (0)	
Gallbladder adenocarcinoma	1 (3.3)	0 (0)	
Colorectal adenocarcinoma	6 (20.0)	1 (4.2)	
Clear cell renal cell carcinoma	1 (3.3)	1 (4.2)	
Uterine leiomyosarcoma	1 (3.3)	0 (0)	
Endometrioid adenocarcinoma	1 (3.3)	0 (0)	
Cervical squamous cell carcinoma	2 (6.7)	2 (8.3)	
Lower extremity epithelioid sarcoma	1 (3.3)	0 (0)	
Nonkeratinizing undifferentiated nasopharyngeal carcinoma	0 (0)	5 (20.8)	
Invasive ductal carcinoma	0 (0)	1 (4.2)	
Laryngeal Squamous Cell Carcinoma	0 (0)	1 (4.2)	
Submandibular gland Polymorphous adenocarcinoma	0 (0)	1 (4.2)	
Gastric adenocarcinoma	0 (0)	1 (4.2)	
**GTV volume**			
Mean	107.7	96.6	0.708
Range	1.3~816.8	3.5~455.5	
**Radiation dose (Gy)**			
Mean	58.2	52.6	0.073
Range	36.0~71.5	30.0~66.0	
**Radiation fraction dose (Gy)**			
Median	4.0	4.0	0.854
Range	3.0~6.0	3.0~7.0	

ECOG, Eastern Cooperative Oncology Group; PS, performance status; GTV, gross tumor volume; CChRT, hypo-fractionated radiotherapy and concurrent chemotherapy; hRT, hypo-fractionated radiotherapy.

**Figure 1:**
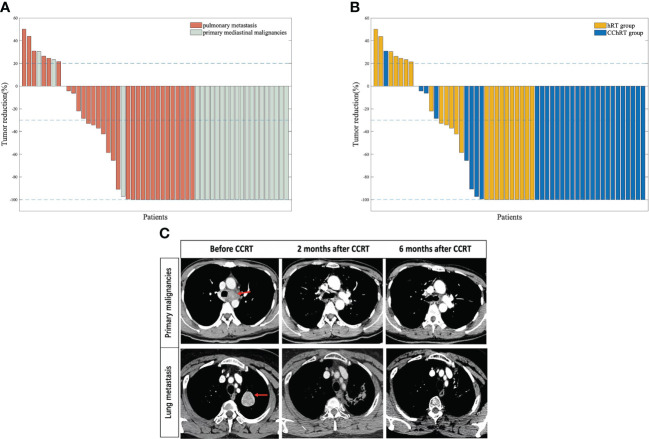
**(A)** Percentage change in tumor size at first follow-up (based on baseline assessment) in patients with oligometastatic pulmonary lesions (n=31) and primary mediastinal malignancies (n=23) **(B)** Percentage change in tumor size at first follow-up (based on baseline assessment) in patients in CChRT group (n=30) and hRT group (n=24). **(C)** A patient with tracheal adenoid cystic carcinoma and a patient with mediastinal metastasis from gallbladder adenocarcinoma were assessed at baseline, 2 months after CCRT and 6 months after CCRT Data cutoff for this analysis was September 2020. Blue lines represent 20% increase and 30% reduction in tumor size, respectively.CChRT, hypo-fractionated radiotherapy and concurrent chemotherapy; hRT, hypo-fractionated radiotherapy.

In the CChRT group, there were 22 patients with CR, 4 patients with PR, 3 patients with SD, and 1 patient with PD (**Table 2**). In the hRT group, there were 10 patients with CR, 5 patients with PR, 2 patients with SD, and 7 patients with PD. The ORR and DCR were 86.7% (26/30) and 96.7% (29/30) in the CChRT group, compared with 62.5% (15/24) and 70.8% (17/24) in the hRT group (*p*=0.033). The median DOR was 19.1 months in CChRT group and 15.9 months in the hRT group, respectively (*p*=0.837). The shrinkage of tumors in the two groups at the first evaluation after treatment had been shown in **Figure 1**.

**Table 2:** Summary of Tumor Responses (n=54).

Response*, n(%)	CChRT group (n=30)	hRT group (n=24)	*P*
CR	22 (73.3)	10 (41.7)	0.033
PR	4 (13.3)	5 (20.8)	
SD	3 (10.0)	2 (8.3)	
PD	1 (3.3)	7 (29.2)	
ORR (CR+PR)	26 (86.7)	15 (62.5)	
DCR (CR+PR+SD)	29 (96.7)	17 (70.8)	

*Tumor response was assessed by RECIST version 1.1.

CR, complete remission; PR, partial remission; SD, stable disease; PD, progressive disease; ORR, overall response rate; DCR, disease control rate; CChRT, hypo-fractionated radiotherapy and concurrent chemotherapy; hRT, hypo-fractionated radiotherapy.

**Figure 2:**
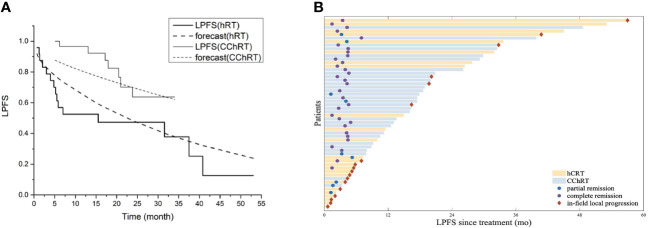
**(A)** Model forecast of in-field LPFS data of CChRT group and hRT group using the LQRGC model (n=54). **(B)** Swimmer plots of in-field LPFS for patients in CChRT group and hRT group (n=54). LPFS, loco-regional progression-free survival; DP, docetaxel-nedaplatin; CChRT, hypo-fractionated radiotherapy and concurrent chemotherapy; hRT, hypo-fractionated radiotherapy; LQRGC, the Linear Quadratic model with effects of four ‘‘R”s, Gompertzian tumor growth and chemotherapeutic agent.

As of September 1st, 2020, the median follow-up time of 25.6 months (range 4.7-68.8 months) for all patients. The median in-field LPFS was 32.8 months in the CChRT group and 15.5 months in the hRT group (*p*=0.003, **Figure 2A**). In-field LPFS rates in the CChRT group were 96.6% at 1 year and 73.4% at 2 years, compared with 52.5% at 1 year and 47.3% at 2 years in the hRT group (**Figure 2A**). The swimmer plots in **Figure 2B** showed individual subject’s pattern of response and in-filed LPFS in two groups.

The acute treatment-related adverse events (AEs) were listed in **Table 3**. The incidences of most toxicities were similar in both groups. The incidence of grade ≥3 esophagitis was 3.3% (1/30) in the CChRT group and 0% (0/24) in the hRT group (*p*=0.555). The incidence of grade ≥3 pneumonitis was 6.7% (2/30) in the CChRT group and 0% (0/24) in the hRT group (*p*=0.343). Most esophageal and pulmonary events were grade 1-2 and did not require medical intervention. 20.0% (6/30) patients in the CChRT group experienced grade ≥3 AEs compared with 16.7% (4/24) patients in the hRT group (*p*=0.754). No patients experienced grade 4 or 5 toxicity.

**Table 3:** Toxicities (n=54).

Toxicities*	CChRT group (n=30)	hRT group (n=24)	
	No. of patients (%)	No. of patients (%)	*P*
**Esophagitis**			
Grade 1-2	23 (76.7)	19 (79.2)	0.555
Grade ≥3	1 (3.3)	0 (0)	
**Pneumonitis**			
Grade 1-2	19 (63.3)	20 (83.3)	0.343
Grade ≥3	2 (6.7)	0 (0)	
**Leukopenia**			
Grade 1-2	15 (50.0)	15 (62.5)	0.528
Grade ≥3	1 (3.3)	1 (4.2)	
**Thrombocytopenia**			
Grade 1-2	6 (20.0)	4 (16.7)	0.703
Grade ≥3	0 (0)	0 (0)	
**Anemia**			
Grade 1-2	11 (36.7)	11 (45.9)	0.293
Grade ≥3	2 (6.7)	3 (12.5)	
**Nausea**			
Grade 1-2	14 (46.7)	8 (33.3)	0.165
Grade ≥3	0 (0)	0 (0)	

*Treatment-related toxicities were assessed by CTCAE version 5.0.

CChRT, hypo-fractionated radiotherapy and concurrent chemotherapy; hRT, hypo-fractionated radiotherapy.

The LQRGC/TCP model with optimized parameters based on hypo-RT schedule (**Supplementary 2.1**) was used in fitting the in-field LPFS data for different histologic subtypes of primary and metastatic thoracic tumors and presented in **Figure 2A**. The values of model parameters and their CIs were detailed in **Table 4**.

**Table 4:** The values of model parameters.

Parameter	Initial LQRGC model		Optimized LQRGC model	
	Value	95%CI	Value	95%CI
*ρ* (cm^-3^)	1.29×10^9^	[1.24×10^9^,1.30×10^9^] [16]	1.37×10^9^	[1.27×10^9^,1.40×10^9^]
*α* (Gy^-1^)	0.07926	[0.07837,0.07934]	0.09065	[0.08874,0.09095]
*β* (Gy^-2^)	0.00755	[0.00752,0.00799]	0.00908	[0.00903,0.00955]
*σ^2^* (Gy^-2^)	0.00106	[8.527×10^-5^,0.00111]	0.00864	[0.00750,0.00873]
*τ_R_* (hour)	4.70	[2.99,4.94]	4.56	[0.65,5.10]
*τ_S_* (hour)	5.94	[2.88,8.36]	5.54	[0.47,6.28]
*T_P_* (day)	105.00	[103.70,126.76]	105.00	[53.73,110.73]
*T_k_* (hour)	260	-	260	-
*K_c_*	1.026×10^9^	[1.022×10^9^,1.11×10^9^]	1.026×10^9^	[1.014×10^9^,1.232×10^9^]
*σ_k_*	3.9×10^8^	[2.84×10^8^,4.00×10^8^]	3.9×10^8^	[2.51×10^8^,4.36×10^8^]
*δ*	0.191	[0.178,0.194]	0.191	[0.131,0.194]
*C_DP_*	0.2	[0.194,0.206]	0.2	[0.175,0.25]
*α/β*(Gy)	10.50	-	9.99	-

LQRGC, the Linear Quadratic model with effects of four ‘‘R”, Gompertzian tumor growth and chemotherapeutic agent established based on NSCLC patients in our previous study (9, 15, 16) and the LQRGC parameters optimized according to hypo-fractionated radiotherapy schedule and α/β values of atypical primary and metastatic thoracic malignancies.

The intermediate items of in-field LPFS calculation, including BED for different histologic subtypes of tumors, were shown in **Supplementary 2.2**. The mean BED of the LQRGC model in the CChRT group was 72.34Gy, compared with 67.25Gy in the hRT group (*p*=0.251). The chemo-induced BED for weekly DP regimen was 2.21Gy. The radio-induced BED was lower in the hRT group (CChRT group: 70.13Gy; hRT group: 67.25Gy, *p*=0.517), which was associated with its lower mean radiation dose.

The quality metrics of the LQRGC model was presented in **Supplementary 2.2**. The average relative and absolute fitting errors for in-field LPFS in the CChRT group were -4.56% and -4.75%, versus 3.36% and 0.44% in the hRT group.

## Discussion

This study compared the efficacy of hypo-RT with concurrent weekly DP with hypo-RT alone for atypical histologic subtypes of primary and metastatic mediastinal malignancies. The ORR was 86.7% (26/30) and DCR was 96.7% (29/30) in CChRT group, while the ORR was 62.5% (15/24) and DCR was 70.8% (17/24) in hRT group (*p*=0.033). The 2-year in-filed LPFS of CChRT and hRT group was 73.4% and 47.3%, respectively (*p*=0.003).

Atypical histologic subtypes of thoracic malignancies, such as thymic carcinoma, thymic carcinoid and tracheal carcinoma, were previously thought to be radioresistant. Surgical resection is the mainstay of treatment and radiation therapy has only been used for inoperable patients with those cancers (18, 19). However, the outcome of radiation therapy was poor, with ORR of about 57.1% and 2-year OS of 21% to 40% (20–22). Jumpei et al. reported median OS of 21.4 months and ORR of 57.1% for 14 patients with thymic carcinoma treated by chemoradiotherapy. The total radiation doses were 31.5-64Gy with 1.8-2.5Gy per fraction and the most common used chemotherapy were etoposide-cisplatin (EP) and regimens consisting of cyclophosphamide, doxorubicin, hydrochloride and vincristine alternating with cisplatin and etoposide (CAV/PVP) (21). Mornex et al. reported 1-year OS of 46% and 2-year OS of 21% for 106 patients with tracheal carcinoma. The total radiation doses were 30-70Gy with 2Gy per fraction. Only 7.5% patients received chemotherapy, with a variety of drug combinations (22). The mechanisms underlying the radio-resistance of these tumors include the differential tissue-specific gene expression (e.g., p53, ataxia telangiectasia mutated status), cell cycle regulation, DNA repair, and tumor angiogenesis after exposure to ionizing radiation (23, 24). The application of hypo-RT and a more potent radiosensitizing chemotherapy regimen has the potential of overcoming their intrinsic radioresistance.

In this study, the ORR of CChRT group and hRT group was 86.7% and 62.5%, both better than historical studies. The superior outcome in this study was firstly attributed to the use of hypo-fractionated IMRT technique. Previous studies indicated hypo-RT acted *via* novel biologic mechanisms and high BEDs, providing a means to overcome radiation resistance (25, 26). Compared to conventional fractionated radiotherapy, hypo-RT improved outcomes by overcoming hypoxia-induced radio-resistance, activating innate and adaptive immunity, and triggering bystander or abscopal tumoricidal effects (27). Our previous study also showed that hypo-fractionated IMRT improved loco-regional control in NSCLC patients treated with an escalated fraction size (16). In Iyengar et al.'s (28) study, hypofractionated IGRT (60 Gy in 15 fractions) showed a trend toward improvement in local recurrence and distant metastasis in NSCLC, accompanied by a statistical increase in grade 2 toxic effects (28). In the current study, for patients at a high risk of toxicities (bulky tumor, or tumor adjacent to esophagus or spinal cord), split-course hypo-RT was delivered with an interval of 4 weeks between two courses and re-planning was performed for the second course, which allowed the recovery of treatment-related adverse events such as esophagitis and lung injury. Secondly, the use of weekly DP further improved the efficacy of hRT in this study. Weekly DP exhibited promising radio-sensitizing effect in esophageal squamous cell carcinoma, and nasopharyngeal carcinoma and NSCLC (10, 29, 30). In our previous study, weekly DP also showed higher chemo-induced BED than 3-week pemetrexed-platinum and weekly paclitaxel-platinum regimens in concurrent chemoradiotherapy (CCRT) for patients with NSCLC (9). It was also noted that weekly DP was well tolerated with low incidence of >=grade 2 toxicities, especially pulmonary toxicity, which is vital for subsequent systemic therapies in patients with metastatic thoracic malignancies.

A recently developed LQRGC model incorporating effects of four “R”s, Gompertzian tumor growth and chemotherapeutic agents (9) was adopted to quantify the effects of CCRT in the current study. The LQRGC model, established based on data of patients with NSCLC, was used to fit the in-field LPFS curves in tumors with different pathologic types and extended to LQRGC with model optimized parameters (**Table 4**). The actual in-field LPFS curves of the two groups (**Figure 2A**) indicated that weekly DP regimen had a good radio-sensitization effect for these thoracic cancers. Quantified by the LQRGC model, the mean BED of CChRT group reached 72.34Gy (**Supplementary 4**), compared with 67.25Gy of hRT group. This explained the improved local control rates of hypo-RT with DP regimen in atypical histologic subtypes of mediastinal malignancies. In our previous study, weekly-DP regimen increased BED by approximately 3.14% in CCRT for non-small cell lung cancer (9). In this study, the chemo-induced BED for weekly DP was 2.21Gy, accounting for 3.05% of total BED, which indicates its similar radio-sensitization effect in atypical primary and secondary mediastinal malignancies.

This study was performed in a heterogeneous population that includes several histologic subtypes of primary and secondary mediastinal cancers. Because of the low incidence, it is difficult to conduct studies for a specific type. We grouped these malignancies together because they are all considered relatively radiation-resistant and face the risk of radiation-induced toxicities to critical organs such as trachea, esophagus and heart. This study demonstrated the efficacy and feasibility of concurrent weekly DP regimen combined with hypo-RT. We admit that the sample size was too small to draw firm conclusions specific to a tumor type. Our TCP model also suffered from its limited applicability due to the radiobiological assumption which has not been confirmed, and the heterogenous model parameters of various histology. This study was also limited by its retrospective nature in which the toxicities might be underestimated. Further studies are warranted to validate our findings.

## Conclusions

Hypo-fractionated radiotherapy and concurrent weekly docetaxel and nedaplatin showed improved efficacy on in-field disease control and good tolerability compared with hypo-fractionated radiotherapy alone in different histologic subtypes of primary and metastatic mediastinal malignancies.

## Data availability statement

The raw data supporting the conclusions of this article will be made available by the authors, without undue reservation.

## Ethics statement

The studies involving human participants were reviewed and approved by the ethics committee of Sun Yat-sen University Cancer Center. The patients/participants provided their written informed consent to participate in this study. Written informed consent was obtained from the individual(s) for the publication of any potentially identifiable images or data included in this article.

## Author contributions

HL, BQ, and FM contributed to conception and design of the study. XA and BW organized the database. XA and YZ performed the statistical analysis and modeling. FM, XA, and BW wrote the first draft of the manuscript. DW, FL, NC, RZ, JG, XH, and SY wrote sections of the manuscript. All authors contributed to manuscript revision, read, and approved the submitted version.

## Funding

This work was supported by the National Natural Science Foundation of China (Grant Number 82073328).

## Conflict of interest

Author YZ was employed by the company Evidance Medical Technologies Inc.

The remaining authors declare that the research was conducted in the absence of any commercial or financial relationships that could be construed as a potential conflict of interest.

## Publisher’s note

All claims expressed in this article are solely those of the authors and do not necessarily represent those of their affiliated organizations, or those of the publisher, the editors and the reviewers. Any product that may be evaluated in this article, or claim that may be made by its manufacturer, is not guaranteed or endorsed by the publisher.
