# Supplementary material for: Evaluation of the efficacy and feasibility of concurrent weekly docetaxel-nedaplatin and hypo-fractionated radiotherapy in atypical histologic subtypes of primary and metastatic mediastinal malignancies
Source: Front Oncol. 2022 Oct 7;12:974394. doi: 10.3389/fonc.2022.974394 (PMC9585306; doi:10.3389/fonc.2022.974394)
Supplement: Supplementary file 1 [file DataSheet_1.docx]

Supplementary Material

# Supplementary Data.

## The model parameters in the LQRGC model.

The survival fraction (SF) of tumor cells in this model is expressed as:

$SF(t)=\exp(-\alpha D-\beta G(\tau_{R})D^{2}+(\frac{1}{2}\sigma^{2})G(\tau_{s})D^{2}+\ln2\frac{T-T_{k}}{T_{P}}+{(\ln2\frac{t}{T_{P}})}^{\delta}-C_{i})$ (Eq. 1)

where *τ_R_* is the average DNA repair time; *τ_S_* is average resensitization time; *σ* is the variance of the Gaussian distribution of random variable *α*, immediately after an acute irradiation; *T* is the overall treatment time; *T_k_* is the delayed time for regrowth; *T_p_* is the cell number doubling time; *t* is the elapsed time since the end of treatment; *δ* refers to the Gompertzian tumor growth after radiation; and *C_i_* represents the overall cell kill effect of the chemotherapeutic regimen. The first and second terms describe cell killing by one-track and two-track action (and possible repair), respectively. The third term refers to intercellular diversity of radiosensitivity and re-sensitization. The fourth term refers to the delayed tumor regrowth. The fifth term involving parameter d refers to the Gompertzian tumor growth after radiation and the last term *C_i_* represents the overall cell kill effect of the chemotherapeutic regimen.

## The model parameters in the TCP formulation

The TCP function derives from Tai et al’s study:

| $TCP(t)=1-\frac{1}{\sqrt{2\pi}}\int_{-\infty}^{x_{0}} \exp(-\frac{x^{2}}{2})dx$ | (Eq. 4) |
| --- | --- |

with

| $x_{0}=\frac{\overline{K}-K_{cr}}{\sigma_{k}}=\frac{SF-K_{cr}/K_{0}}{\sigma_{k}/K_{0}}$ | (Eq. 5) |
| --- | --- |

*σ_k_* is the Gaussian width for the distribution of tumour cell numbers, *K_0_* is the initial average number of clonogenic cells and *K_cr_* is a critical number under which the tumor would be controlled for an individual patient. *K_0_*=*V·ρ*, where *V* is the tumor volume (GTV volume) and *ρ* is the number density of the clogenic tumor cells (#/cm^3^).

# Supplementary Figures and Tables

## Supplementary Figures


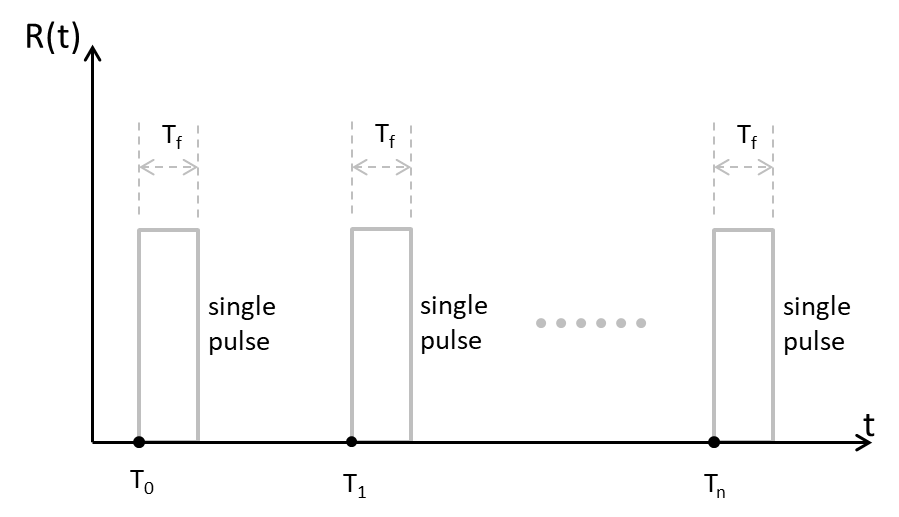


**Supplementary Figure 1.** Pulse train of the hypo-fractionated dose schedule in LQRGC model.

Abbreviations: LQRGC, the Linear Quadratic model with effects of four ‘‘R”, Gompertzian tumor growth and chemotherapeutic agent established based on NSCLC patients in our previous study [9,15,16] and the LQRGC parameters optimized according to hypo-fractionated radiotherapy schedule and *α/β* values of atypical primary and metastatic thoracic malignancies; CChRT, hypo-fractionated radiotherapy and concurrent chemotherapy; hRT, hypo-fractionated radiotherapy; BED: biologically effective dose;

## Supplementary Tables

**Supplementary Table 1.** [BED result](http://www.baidu.com/link?url=jz9bLo3P8bO8d_YwoVygYBchPgUgC9-SbMQ80H0T_ipQCH_6FzNlX_OE9iyemL534NnEQEc8YfmesIqqGHVTd_sUulZIY0bc-dAkazHD_rxmJ8H7ybxEtv7vln58OuWP)s of LQRGC/TCP Model.

| Parameter | CChRT group | hRT group |
| --- | --- | --- |
| Mean BED | 72.34 | 67.25 |
| Radio-induced BED | 70.13 | 67.25 |
| $D$ | 58.25 | 52.57 |
| $\beta G\left(\tau_{R}\right)D^{2}/\alpha$ | 23.64 | 28.61 |
| $-\frac{1}{2}\sigma^{2}G\left(\tau_{S}\right)D^{2}/\alpha$ | -11.37 | -13.78 |
| $-\ln2\frac{T-T_{k}}{T_{P}}/\alpha$ | -0.38 | -0.16 |
| $C/\alpha$ | 2.21 | 0 |

Abbreviations: LQRGC, the Linear Quadratic model with effects of four ‘‘R”, Gompertzian tumor growth and chemotherapeutic agent established based on NSCLC patients in our previous study [9,15,16] and the LQRGC parameters optimized according to hypo-fractionated radiotherapy schedule and *α/β* values of atypical primary and metastatic thoracic malignancies; CChRT, hypo-fractionated radiotherapy and concurrent chemotherapy; hRT, hypo-fractionated radiotherapy; BED: biologically effective dose;

**Supplementary Table 2.** Model Quality Metrics.

|  | CChRT group | hRT group |
| --- | --- | --- |
| Average relative error | -4.56% | 3.36% |
| Average absolute error | -4.75% | 0.44% |

Abbreviations: CChRT, hypo-fractionated radiotherapy and concurrent chemotherapy; hRT, hypo-fractionated radiotherapy;
